# Protective Effect of Quercetin against H_2_O_2_-Induced Oxidative Damage in PC-12 Cells: Comprehensive Analysis of a lncRNA-Associated ceRNA Network

**DOI:** 10.1155/2020/6038919

**Published:** 2020-12-01

**Authors:** Zheyu Zhang, Pengji Yi, Min Yi, Xiaoliang Tong, Xin Cheng, Jingjing Yang, Yang Hu, Weijun Peng

**Affiliations:** ^1^Department of Integrated Traditional Chinese & Western Medicine, The Second Xiangya Hospital, Central South University, Changsha, Hunan 410011, China; ^2^Department of Gastroenterology, Xiangya Hospital, Central South University, Changsha, Hunan 410008, China; ^3^Department of Gastroenterology, Affiliated Hospital of Guilin Medical University, Guilin, Guangxi 541001, China; ^4^Department of Dermatology, Third Xiangya Hospital, Central South University, Changsha 410013, China; ^5^Department of Integrated Traditional Chinese and Western Medicine, Xiangya Hospital, Central South University, Changsha, Hunan 410008, China

## Abstract

Quercetin is a bioflavonoid with potential antioxidant properties. However, the mechanisms underlying its effects remain unclear. Herein, we focused on integrating long noncoding RNA (lncRNA), microRNA (miRNA), and messenger RNA (mRNA) sequencing of PC-12 cells treated with quercetin. We treated PC-12 cells with hydrogen peroxide to generate a validated oxidative damage model. We evaluated the effects of quercetin on PC-12 cells and established the lncRNA, miRNA, and mRNA profiles of these cells. Gene Ontology and Kyoto Encyclopedia of Genes and Genomes analyses of these RNAs were conducted to identify the key pathways. Quercetin significantly protected PC-12 neuronal cells from hydrogen peroxide-induced death. We identified 297, 194, and 14 significantly dysregulated lncRNAs, miRNAs, and mRNAs, respectively, associated with the antioxidant effect of quercetin. Furthermore, the phosphatidylinositol-3-kinase/protein kinase B pathway was identified as the crucial signalling pathway. Finally, we constructed a lncRNA-associated competing endogenous RNA (ceRNA) network by utilizing oxidative damage mechanism-matched miRNA, lncRNA, and mRNA expression profiles and those changed by quercetin. In conclusion, quercetin exerted a protective effect against oxidative stress-induced damage in PC-12 cells. Our study provides novel insight into ceRNA-mediated gene regulation in the progression of oxidative damage and the action mechanisms of quercetin.

## 1. Introduction

The maintenance of normal physiological functions depends on the balance between reactive oxygen species (ROS) and intracellular antioxidant factors. Oxidative stress (OS), caused by the imbalanced regulation of oxidant and antioxidant systems in cells, is deleterious to cells through DNA damage, cellular dysfunction, and apoptosis [1, 2]. OS has been implicated in the pathogenesis of numerous disorders by affecting the normal functions of several tissues [3–5]. Furthermore, numerous age-related and neurodegenerative diseases, such as Alzheimer's disease (AD), Parkinson's disease (PD), and inherited mitochondrial disorders, are also directly associated with OS [6–8]. Considering this relationship, extensive research is focused on uncovering the underlying mechanisms and role of OS in the onset and development of disease. It is thought that the discovery of new therapeutic strategies for alleviating OS may be helpful for the treatment of many diseases, especially neurodegenerative diseases.

Quercetin (QUE) is a natural flavonoid widely distributed in many fruits and vegetables. It has numerous biological activities, including antitumour effects, anti-inflammatory properties, cardiovascular protection, and regulation of blood sugar levels [9]. An increasing body of evidence indicates that QUE has the ability to scavenge ROS, such as superoxide radical anion and hydroxyl radical, and can act as an antioxidant agent. Previous in vitro research studies suggested that QUE has the ability to quench oxygen free radicals [10–12]. Recent findings indicated that QUE can provide protection against OS when administered in vivo [13, 14]. In addition, several studies revealed signal transduction pathways associated with the ability of QUE to counteract oxidative damage, such as activation of the nuclear factor erythroid 2-related factor 2-antioxidant response element (Nrf2-ARE) pathway, induction of Nrf2 nuclear translocation, and an increase in glutathione levels [15–17]. To date, research studies on the antioxidant mechanism of QUE are insufficient.

Recently, noncoding RNAs (ncRNAs), including long ncRNAs (lncRNAs; with lengths > 200 bp) and microRNAs (miRNAs; measuring < 200 nucleotides long), have attracted considerable attention. Dysregulation of lncRNAs and miRNAs has been strongly associated with the pathogenesis of OS [18–20]. Researchers have theorized that lncRNAs harbouring miRNA response elements could compete with each other for binding to a common miRNA, thereby regulating the levels and downstream functions of miRNAs. This hypothesis is termed “competing endogenous RNA (ceRNA)” [21]. Previous evidence demonstrated that OS is strongly correlated with ceRNA. lncRNA LINC01619, as the endogenous competitor, has been shown to regulate miR-27a/forkhead box protein O1 (miR-27a/FOXO1) for the induction of endoplasmic reticulum stress and podocyte damage and subsequently trigger OS [22]. Likewise, by competing to bind to miRNA-150, lncRNA FOXD3-AS1 blocked its protective effect, thereby contributing to the OS-induced apoptosis of lung epithelial cells [23]. Overall, these studies indicated that the link between ncRNAs and OS is stronger than previously thought. However, there is limited knowledge on the role of functional RNA molecules and RNA-mediated regulation networks in the antioxidant mechanism of QUE. Thus, further efforts (i.e., analysis of lncRNA and miRNA) to elucidate the antioxidant mechanisms of QUE are warranted.

The cell line rat pheochromocytoma PC-12 has been commonly used in the investigation of neurological diseases, such as AD and PD [24]. In the present study, a model of oxidative injury was established by hydrogen peroxide- (H_2_O_2_-) induced damage in PC-12 cells [25] and used to identify the antioxidant function of QUE. Furthermore, transcriptomics and bioinformatics analyses were conducted to further investigate the regulatory mechanisms of QUE in protecting neuronal cells from oxidative damage.

## 2. Materials and Methods

### 2.1. Cell Culture and Treatment

PC-12 cells derived from a transplantable rat pheochromocytoma were cultured in Dulbecco's modified Eagle's medium containing 10% foetal calf serum, 100 U/mL penicillin, and 100 *μ*g/mL streptomycin at a density of 1 × 10^4^ cells per well (5% CO_2_ at 37°C). Experiments were performed 24 h after cells were seeded. The PC-12 cells were pretreated with various concentrations (6.25, 12.5, or 25 *μ*M) of QUE in serum-free Dulbecco's modified Eagle's medium for 2 h. Subsequently, the pretreated cells were cultured for an additional 6 h with 500 *μ*M H_2_O_2_ to stimulate oxidative damage, as previously described [24]. Untreated cells and cells treated with H_2_O_2_ alone were used as normal and model control, respectively. H_2_O_2_ was purchased from Sigma-Aldrich (St. Louis, MO, USA).

### 2.2. Cell Viability Assay

Cellular toxicities of H_2_O_2_ and the protective effect of QUE were measured in PC-12 cells using the MTS assay (Promega Corporation, Madison, WI, USA). After treatment with the indicated concentrations of H_2_O_2_ or QUE, 10 *μ*L MTS kit solution was added, and the cells were incubated for 1 h at 37°C. Subsequently, the absorbance was measured using a Microplate Reader (BioTek Instruments, Winooski, VT, USA).

### 2.3. Biochemical Analysis

Cold phosphate-buffered saline was used to collect treated and untreated PC-12 cell samples through centrifugation according to the instructions provided in each enzyme kit. The cells were homogenized with cold phosphate-buffered saline, and commercial kits (Jiangsu Feiya Biological Technology Co., Ltd.) were used to obtain the supernatant for the measurement of malondialdehyde (MDA), glutathione (GSH), and superoxide dismutase (SOD) activity.

### 2.4. Cell Apoptosis Assay

The protective effect of QUE on apoptosis in PC-12 cells was determined using the Hoechst assay. The cells pretreated with 25 *μ*M QUE were used as the protection group. After treatment of the cells, 5 *μ*g/mL Hoechst staining solution was added to avoid light staining, and the cells were placed under an Olympus fluorescence microscope for imaging. This experiment was repeated thrice.

### 2.5. Western Blotting Analysis

Total protein was extracted from PC-12 cells according to the instructions provided by the manufacturer for the radioimmunoprecipitation assay lysis buffer. The protein assay reagent kit was used to measure the concentrations of the extracted protein. The western blotting protocol was described in detail in our previous study [26]. The glyceraldehyde-3-phosphate dehydrogenase antibody (GAPDH) and *β*-acting were used as the internal reference, and the grey value was analyzed using the ImageJ software.

### 2.6. RNA Extraction and Monitoring of Quality

We selected three groups of PC-12 cells, H_2_O_2_-induced PC-12 cells, and H_2_O_2_-induced PC-12 cells pretreated with 25 *μ*M QUE for RNA sequencing. For the extraction of total cell RNA from PC-12 cells, the Trizol reagent (Invitrogen, Carlsbad, CA, USA) was used in accordance with the protocol provided by the manufacturer. The detection of RNA quality was performed using the following methods: NanoDrop detection, Qubit 2.0 detection, and Agilent 2100 bioanalyzer test.

### 2.7. Small RNA Library Construction, High-Throughput Sequencing, and Data Processing

After passing the sample test, 1.5 *μ*g was used as the starting amount of the RNA sample. The volume was adjusted to 6 *μ*L with water, and the small RNA Sample Prep Kit was used for the construction of the library. Next, the concentration and insert size of the library were determined using Qubit 2.0 and Agilent 2100 bioanalyzer test, respectively. The effective concentration of the library was accurately quantified using quantitative polymerase chain reaction (qPCR). Subsequently, the Illumina NovaSeq platform was used for high-throughput sequencing with a single-end 50-nucleotide read length. The raw image data files obtained by Illumina NovaSeq platform sequencing were converted into raw sequencing sequences (Raw Reads) in a FASTQ file format. The following quality control was performed on the Raw Reads to obtain the high-quality sequences (Clean Reads) by removing the following: (1) for each sample, the series with low-quality values; (2) reads with unknown base N (N is an unrecognizable base) with content ≥ 10%; (3) reads without 3′ linker sequence and inserts; (4) reads contaminated with 5′ joints; (5) the 3′ linker sequence; (6) reads from ploy A/T/C/G; and (7) sequences < 18 or >30 nucleotides.

### 2.8. lncRNA Library Construction, High-Throughput Sequencing, and Data Processing

After the qualification of the samples, library construction was performed. Firstly, the ribosomal RNA (rRNA) of the sample was removed using the epicentre Ribo-Zero™ kit; rRNA-depleted RNA was randomly interrupted by adding fragmentation buffer; rRNA-depleted RNA was used as a template, and random hexamers were used to synthesize the first one cDNA strand. Subsequently, we added buffer, deoxyadenosine triphosphate, deoxyuridine triphosphate, deoxycytidine triphosphate, deoxyguanosine triphosphate, RNase H, and DNA polymerase I to synthesize a second cDNA strand and used AMPure XP beads to purify the cDNA. The purified double-stranded cDNA was then subjected to end repair, and a sequencing adapter was connected. AMPure XP beads were used to select the fragment size. Finally, the U-chain was degraded, and the cDNA library was enriched by PCR. The original sequence (measured by the deribosomal library) was quality controlled, and the method was based on previous studies [27].

### 2.9. Validation through Real-Time qPCR

Real-time qPCR analysis was used to validate the results of RNA-Seq. The ViiA 7 Real-Time PCR System (Applied Biosystems) and 2X PCR master mix (Arraystar, USA) were selected according to the instructions provided by the manufacturer. The lncRNA, mRNA, and miRNA expression levels were normalized to those of *β*-actin, ACTB, and U6, respectively. The details of the primers are shown in Table 1. The data represent the average of three experiments.

### 2.10. Gene Ontology (GO) and Kyoto Gene and Genomic Encyclopedia (KEGG) Pathway Analyses

Furthermore, transcriptional changes were determined at the overall level by GO and KEGG pathway enrichment analyses on mRNAs, target genes of miRNAs, and target genes of lncRNAs related to QUE treatment. Both analyses were conducted using the R language (version 3.5.3).

### 2.11. Construction of the lncRNA-Associated ceRNA Network

The ceRNA hypothesis reveals a novel regulatory mechanism between ncRNAs and coding RNAs [27]. The lncRNA-associated ceRNA network was established following these three steps: (1) lncRNA, mRNA, and miRNA related to OS and those altered by QUE were selected; (2) miRanda and TargetScan were applied to predict the correlation of miRNA with ceRNA (herein, lncRNA and mRNA); and (3) a lncRNA-miRNA-mRNA network was constructed using the Cytoscape v3.01 software.

### 2.12. Statistical Analysis

The SPSS version 22.0 (IBM Corp., Armonk, NY, USA) software was used for statistical analysis in our study. *p* values < 0.05 denoted statistically significant differences. The DEseq software was utilized to process the sequencing data. Using *p* < 0.01 and ∣log_2_(fold change) | >2 as the criteria, the differentially expressed (DE) lncRNAs, DE miRNAs, and DE mRNAs were identified.

## 3. Results

### 3.1. QUE Protected PC-12 Cells from H_2_O_2_-Triggered Injury

**Figure 1:**
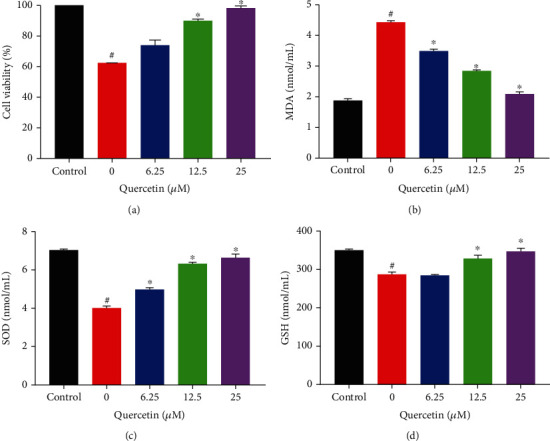
Protective effect of QUE on H_2_O_2_-induced cytotoxicity in PC-12 cells. (a) Effect of QUE on PC-12 cell viability rate induced by H_2_O_2_. The percentage of cell viability was relative to that of untreated control cells. Effect of QUE on (b) MDA, (c) SOD, and (d) GSH in PC-12 cells induced by H_2_O_2_. In the protected group, PC-12 cells were pretreated with different concentrations of QUE for 2 h and subsequently incubated with 500 *μ*M H_2_O_2_ for an additional 6 h. Values represent mean ± SEM of three independent experiments. ^#^*p* < 0.05 versus control; ^∗^*p* < 0.05 versus H_2_O_2_-treated cells. H_2_O_2_: hydrogen peroxide; QUE: quercetin; SEM: standard error of the mean.

The protective effect of QUE on PC-12 cell viability rate induced by H_2_O_2_ was detected by the MTS assay. As shown in Figure 1(a), the cell survival rate in the model group was significantly reduced compared with that noted in the control group, indicating that H_2_O_2_ induced cytotoxic damage to PC-12 cells (*p* < 0.05). Through treatment with QUE, the survival rate of PC-12 cells was significantly increased in a dose-dependent manner (*p* < 0.05).

The protective effect of QUE on the activities of SOD and GSH and the content of MDA in PC 12 cells injured by H_2_O_2_ were detected. As shown in Figure 1(b), the expression levels of MDA in the model group were higher than those recorded in the control group. Compared with the model group, the levels of MDA enzymes in the samples pretreated with QUE were significantly lower. As shown in Figures 1(c) and 1(d), the expression levels of SOD and GSH in the model group were significantly reduced compared with those noted in the control group. Through treatment with QUE, their expression levels were significantly increased in a dose-dependent manner. These results indicate that QUE pretreatment can significantly reduce the inhibitory effect of H_2_O_2_ on antioxidant enzymes.

### 3.2. QUE Protected PC-12 Cells from H_2_O_2_-Induced Cell Apoptosis

**Figure 2:**
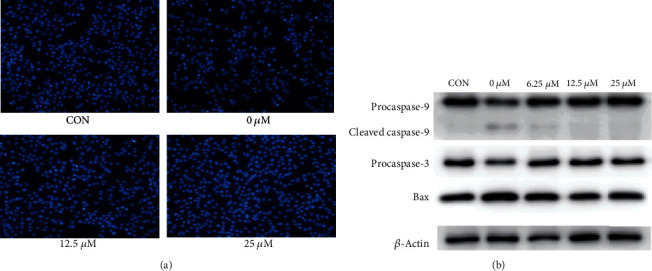
Protective effect of QUE against H_2_O_2_-induced apoptosis in PC-12 cells. (a) The effect of QUE on H_2_O_2_-induced apoptosis detected by Hoechst 33342 staining. (b) Western blotting analysis of the expression of apoptotic proteins. The expression levels of Bax, procaspase-9, cleaved caspase-9, and procaspase-3 were detected. In the protected group, PC-12 cells were pretreated with different concentrations of QUE for 2 h and subsequently incubated with 500 *μ*M H_2_O_2_ for an additional 6 h. H_2_O_2_: hydrogen peroxide; QUE: quercetin.

To investigate the effects of QUE on the H_2_O_2_-induced apoptosis of PC-12 cells, the Hoechst 33342 staining method was used to detect cell apoptosis, as shown in Figure 2(a). The cells in the control group were dark blue. In the model group, the apoptosis of stained cells was significantly increased after induction treatment and appeared blue-white, while the nuclei showed irregular marginalization. In the protective group pretreated with QUE, the phenomenon of cell apoptosis was obviously alleviated.

Furthermore, western blotting was performed to detect the expression of apoptosis-related proteins, including Bax, procaspase-9, cleaved caspase-9, and procaspase-3. As shown in Figure 2(b), compared with the control group, H_2_O_2_ induced an obvious increase in the expression of Bax and cleaved caspase-9, whereas it decreased the expression of procaspase-3 and procaspase-9. Pretreatment with QUE enhanced the expression of procaspase-3 and procaspase-9, whereas it reduced that of Bax and cleaved caspase-9 compared with the H_2_O_2_ group. These results indicate that pretreatment with QUE can significantly protect PC-12 cells from H_2_O_2_-induced apoptosis.

### 3.3. DE mRNA and Functional Enrichment Analysis

**Figure 3:**
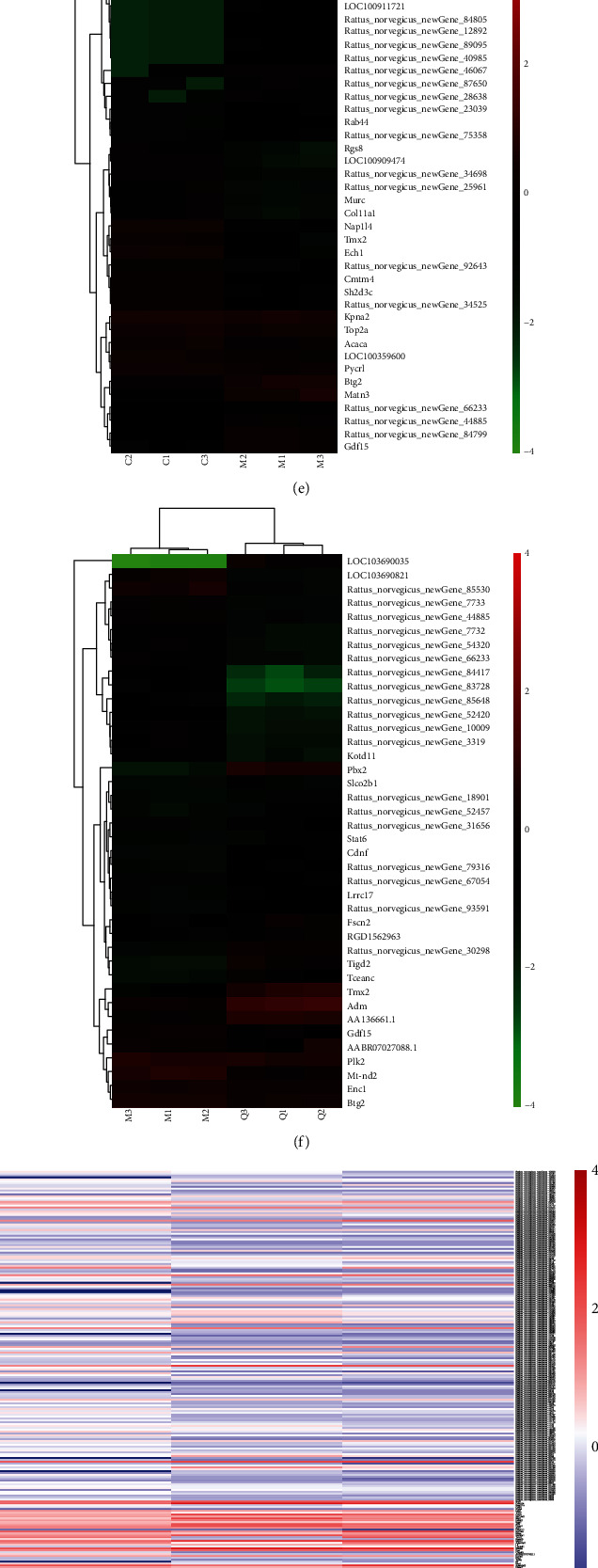
Distinct expression pattern of mRNAs and functional enrichment analysis. The MA plot of DE mRNA expression profiles (a) between the CON group and the model group and (b) between the model group and the QUE group. The volcano plot of DE mRNA expression profiles (c) between the CON group and the model group and (d) between the model group and the QUE group. Unsupervised clustering analysis showing the expression profiles of the top 20 upregulated and downregulated mRNAs (e) between the CON group and the model group and (f) between the model group and the QUE group. (g) Heat map showing 294 mRNAs that were changed when PC-12 cells were subjected to oxidative damage; pretreatment with QUE reversed these alterations. (h) GO enrichment analysis of mRNAs altered by QUE. The superscripted number represents the number of genes annotated in the GO term, the ordinate represents the GO term, and the abscissa represents the −log_10_(*p* value). (i) KEGG enrichment analysis of mRNA altered by QUE. The ordinate represents the KEGG pathway, and the abscissa represents the adjusted *p* value. CON: control; DE: differentially expressed; GO: Gene Ontology; H_2_O_2_: hydrogen peroxide; KEGG: Kyoto Gene and Genomic Encyclopedia; MOL: model; mRNA: messenger RNA; QUE: quercetin.

We firstly focused on changes in the expression of the coding genes. Using the criterion of *p* < 0.01 and ∣log_2_(fold change) | >2, 966 DE genes (i.e., 823 upregulated and 143 downregulated) between the model and control groups and 495 DE genes (i.e., 130 upregulated and 365 downregulated) between the model and QUE groups were detected. The MA plot (Figures 3(a) and 3(b)) and volcano plot (Figures 3(c) and 3(d)) were applied to exhibit differential gene expression in the above two pairs. The expression profiles of the top 20 upregulated and downregulated DE mRNAs are shown in Figures 3(e) and 3(f), using unsupervised clustering analysis. Furthermore, a total of 294 mRNAs related to the protection of neuronal cells from OS through treatment with QUE were identified by comparing gene expression in the three groups (Figure 3(g)). Notably, 278 mRNAs were upregulated in the H_2_O_2_ group compared with the control group. Interestingly, intervention with QUE could reverse these H_2_O_2_-induced alterations, producing expression levels similar to those observed in the control group. Likewise, 16 mRNAs were downregulated in the model group compared with the control group; these changes were also reversed after treatment with QUE. Collectively, these results suggested that OS induced transcriptome alterations in neuronal cells, whereas treatment with QUE could reverse these changes to a large extent.

GO and KEGG analyses were performed to investigate the function of QUE-targeted mRNAs. As shown in Figure 3(h), the most enriched biological process (BP) in GO terms were lymphocyte chemotaxis, macrophage chemotaxis, cell proliferation, etc. The most enriched cellular component (CC) terms were acrosomal vesicle, extracellular space, nucleoplasm, etc., while the most enriched molecular function (MF) terms were chemokine activity, serine-type endopeptidase, ligase activity, etc. The top significantly enriched KEGG pathways are shown in Figure 3(i). The tumour necrosis factor signalling pathway, FOXO signalling pathway, and mitogen-activated protein kinase signalling pathway may be closely involved in the protective effect to neuronal cells against OS.

### 3.4. DE lncRNAs and Functional Enrichment Analysis

**Figure 4:**
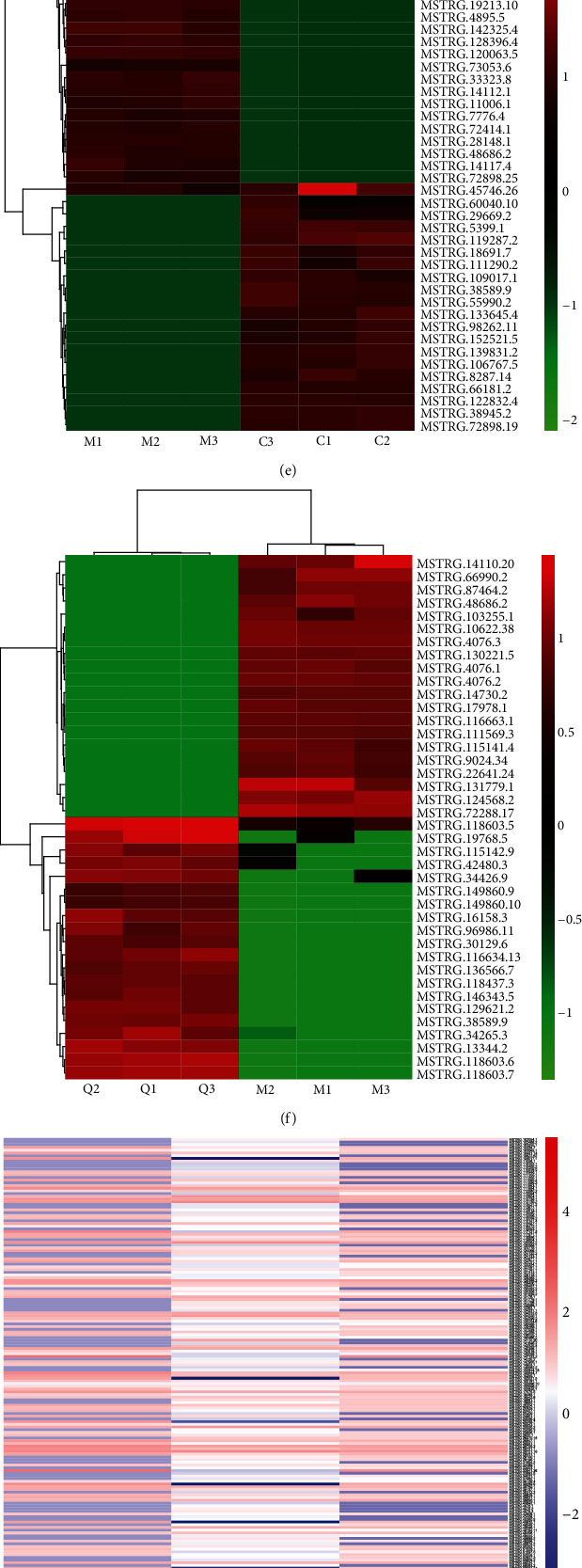
Distinct expression pattern of lncRNAs and functional enrichment analysis. The MA plot of the expression profiles of DE lncRNAs (a) between the CON group and the model group and (b) between the model group and the QUE group. The volcano plot of the expression profiles of DE lncRNAs (c) between the CON and model groups and (d) between the model and QUE groups. Unsupervised clustering analysis showing the expression profiles of the top 20 upregulated and downregulated lncRNAs (e) between the CON and model groups and (f) between the model and QUE groups (f). (g) Heat map showing 197 lncRNAs that were altered when PC-12 cells were subjected to oxidative damage; pretreatment with QUE reversed these alterations. (h) GO enrichment analysis of targeted genes for lncRNAs altered by QUE. The superscripted number represents the number of genes annotated in the GO term, the ordinate represents the GO term, and the abscissa represents the −log_10_(*p* value). (i) KEGG enrichment analysis of targeted genes for lncRNAs altered by QUE. The ordinate represents the KEGG pathway, and the abscissa represents the adjusted *p* value. CON: control; DE: differentially expressed; GO: Gene Ontology; H_2_O_2_: hydrogen peroxide; KEGG: Kyoto Gene and Genomic Encyclopedia; lncRNA: long noncoding RNA; MOL: model; QUE: quercetin.

Likewise, the MA plot (Figures 4(a) and 4(b)) and volcano plot (Figures 4(c) and 4(d)) were applied to compare the DE lncRNA expression profiles of “model group vs. control group” and “QUE group vs. model group.” Like mRNA, the expression profiles of the top 20 upregulated and downregulated DE lncRNAs are exhibited in Figures 4(e) and 4(f). Using the same criterion (fold change ≥ 2 and *p* < 0.01), 1,186 DE lncRNAs (i.e., 1,051 upregulated and 135 downregulated) in the control group compared with the model group and 489 DE lncRNAs (i.e., 228 upregulated and 261 downregulated) in the model group compared with the QUE treatment group were detected. In comparison with the control group, 197 lncRNAs were clearly dysregulated in the model group (i.e., 184 upregulated and 13 downregulated) (Figure 4(g)). Moreover, after treatment with QUE, these H_2_O_2_-induced lncRNA alterations were reversed, producing expression levels similar to those noted in the control group. We subsequently performed a functional enrichment analysis using these 197 lncRNAs.

As shown in Figure 4(h), GO analysis regarding QUE-targeted lncRNA potential target genes indicated that the most enriched GO terms included neutrophil chemotaxis, lymphocyte chemotaxis, and macrophage chemotaxis (BP); extracellular space and acrosomal vesicle (CC); and chemokine activity, serine-type endopeptidase inhibitor activity, and ligase activity (MF). As shown in Figure 4(i), the PI3K-AKT signalling pathway, interleukin 17 (IL17) signalling pathway, and neuroactive ligand-receptor interaction were enriched in the KEGG analysis of lncRNA potential target genes.

### 3.5. DE miRNAs and Functional Enrichment Analysis

**Figure 5:**
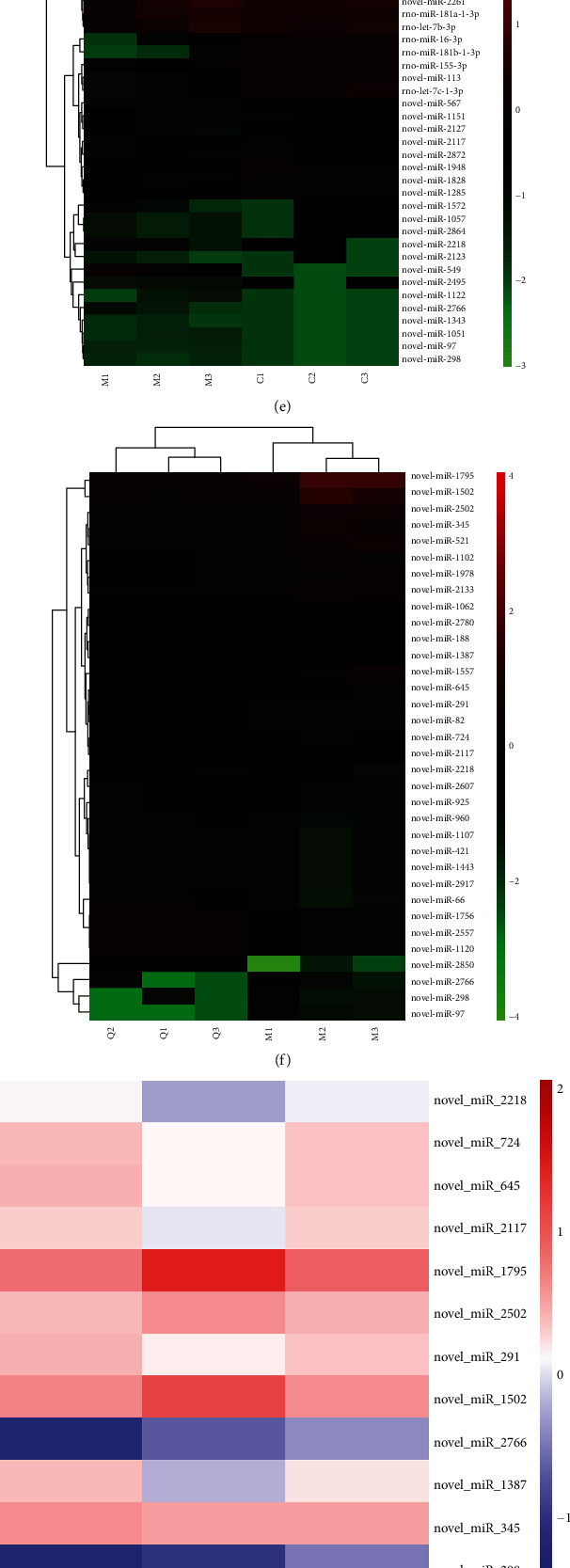
Distinct expression pattern of miRNAs and functional enrichment analysis. The MA plot of the expression profiles of DE miRNAs (a) between the CON and model groups and (b) between the model and QUE groups. The volcano plot of the expression profiles of DE miRNAs (c) between the CON and model groups and (d) between the model and QUE groups. Unsupervised clustering analysis showing the expression profiles of the top 20 upregulated and downregulated miRNAs (e) between the CON and model groups and (f) between the model and QUE groups. (g) Heat map showing 14 miRNAs that were altered when PC-12 cells were subjected to oxidative damage; pretreatment with QUE reversed these alterations). (h) GO enrichment analysis of targeted genes for miRNAs altered by QUE. The superscripted number represents the number of genes annotated in the GO term, the ordinate represents the GO term, and the abscissa represents the −log_10_(*p* value). (i) KEGG enrichment analysis of targeted genes for miRNAs altered by QUE. The ordinate represents the KEGG pathway, and the abscissa represents the adjusted *p* value. CON: control; DE: differentially expressed; GO: Gene Ontology; H_2_O_2_: hydrogen peroxide; KEGG: Kyoto Gene and Genomic Encyclopedia; miRNA: microRNA; MOL: model; QUE: quercetin.

By comparing the miRNA transcriptomes from different groups, 135 H_2_O_2_-responsive miRNAs (i.e., 122 upregulated and 13 downregulated in the H_2_O_2_ intervention group) and 34 QUE-responsive miRNAs (i.e., five upregulated and 29 downregulated after treatment with QUE) were identified. The MA plot (Figures 5(a) and 5(b)) and volcano plot (Figures 5(c) and 5(d)) were applied to detect H_2_O_2_-responsive miRNAs and QUE-responsive miRNAs. The results of the unsupervised clustering analysis, indicating the top 20 upregulated and downregulated DE miRNAs, are shown in Figures 5(e) and 5(f). Compared with the control group, 14 miRNAs in the model group were upregulated, and this effect was reversed after treatment with QUE. Of note, all those miRNAs were newly discovered. This effect was reversed after treatment with QUE (Figure 5(g)). Functional enrichment analysis was subsequently performed to investigate the functions of these dysregulated miRNAs based on their target genes.

In the GO analysis (Figure 5(h)), the top two terms in BP were negative regulation of transcription from the RNA polymerase II promoter and positive regulation of transcription from the RNA polymerase II promoter. MFs were mainly enriched in the transcription factor complex and transcription repressor complex. Regarding CCs, the enriched terms included sequence-specific DNA binding and transcription factor activity, and sequence-specific DNA binding. The most enriched KEGG pathways are shown in Figure 5(i). Major genes were enriched in the following pathways: Hippo signalling pathway, Wnt signalling pathway, etc. These results suggest that miRNA-regulated target mRNAs may participate in the protective function of QUE through these pathways. The PI3K-AKT signalling pathway has also been found to be closely related to this process.

### 3.6. Expression Profile Validation

**Figure 6:**
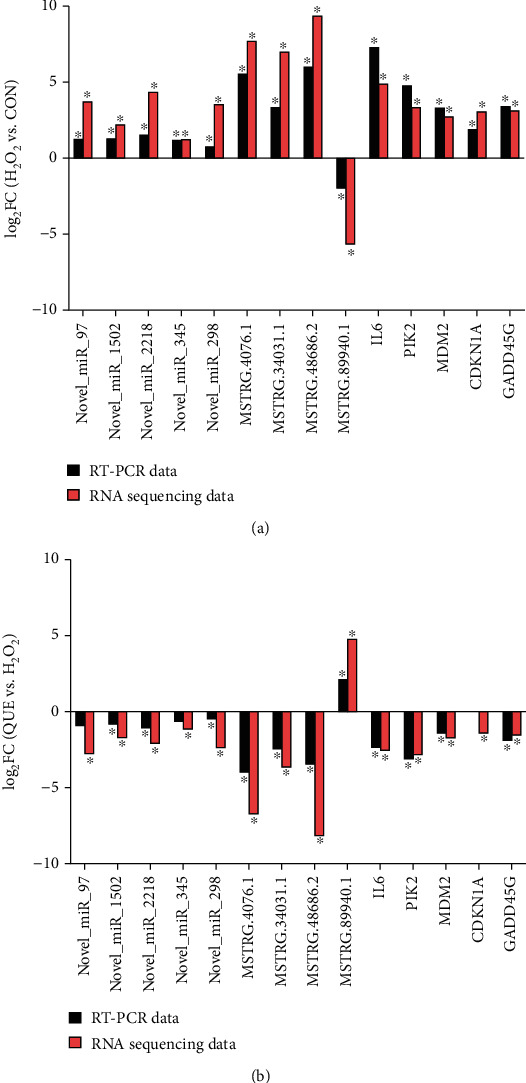
Differential expression of ncRNAs and mRNAs validated by real-time quantitative polymerase chain reaction (qPCR). The data showed that the expression levels of the lncRNAs (MSTRG.4076.1, MSTRG.48686.2, and MSTRG.34031.1), miRNAs (novel_miR_97, novel_miR_298, novel_miR_2218, novel_miR_1502, and novel_miR_2117), and mRNA (PLK2, MDM2, IL6, GADD45G, and CDKN1A) were upregulated in H_2_O_2_-induced PC-12 cells and downregulated when pretreated with QUE. The expression levels of MSTRG.89940.1 were downregulated in H_2_O_2_-induced PC-12 cells and upregulated when pretreated with QUE. The real-time qPCR results were consistent with the RNA sequencing data. ^∗^*p* < 0.05, (a) H_2_O_2_ vs. CON; (b) QUE vs. H_2_O_2_. QUE represents H_2_O_2_-induced PC-12 cells pretreated with QUE. CON represents untreated PC-12 cells. H_2_O_2_ represents H_2_O_2_-induced PC-12 cells. CON: control; H_2_O_2_: hydrogen peroxide; lncRNA: long noncoding RNA; mRNA: messenger RNA; miRNA: microRNA; ncRNA: noncoding RNA; QUE: quercetin.

We conducted real-time qPCR analysis to verify the findings obtained from RNA-Seq. Of the DE mRNAs most relevant to the antioxidant stress mechanism of QUE, five mRNAs enriched in the FOXO signalling pathway were selected for real-time qPCR: polo-like kinase 2 (PLK2), MDM2, IL6, growth arrest and DNA damage-inducible gamma (GADD45G), and cyclin-dependent kinase inhibitor 1A (CDKN1A). Furthermore, nine dysregulated ncRNAs were selected for real-time qPCR analysis, including five miRNAs (i.e., novel_miR_97, novel_miR_298, novel_miR_2218, novel_miR_1502, and novel_miR_2117) and four lncRNAs (i.e., MSTRG.89940.1, MSTRG.4076.1, MSTRG.48686.2, and MSTRG.34031.1). As shown in Figure 6, the expression levels determined by real-time qPCR were in agreement with the results of RNA-Seq. Thus, all lncRNAs, miRNAs, and mRNAs were affirmed as targets closely related to treatment with QUE and included in further analyses.

### 3.7. QUE Activates the PI3K-AKT Pathway in Oxidatively Damaged PC-12 Cells

**Figure 7:**
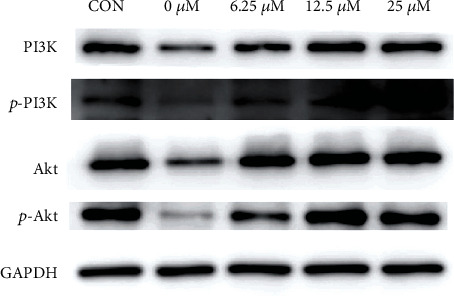
QUE activates the PI3K-AKT pathway in oxidatively damaged PC-12 cells. PC-12 cells were pretreated with different concentrations of QUE for 2 h, and the expression of proteins in the PI3K-AKT signalling pathway was detected through western blotting analysis. GADPH was used as a loading control. GADPH: glyceraldehyde-3-phosphate dehydrogenase; QUE: quercetin.

By combining our findings of the KEGG analysis and those of previous studies, we hypothesized that the PI3K-AKT pathway may be a key pathway for the antioxidant mechanism of QUE. As shown in Figure 7, the protein levels of p-PI3K, p-AKT, PI3K, and AKT were increased in the QUE group, in a concentration-dependent manner, compared with those recorded in the model group. This result suggested that the protein levels of p-PI3K, p-AKT, PI3K, and AKT were increased in response to QUE. The above results confirm that QUE may protect PC-12 cells from H_2_O_2_-induced oxidative damage by activating the PI3K-AKT signalling pathway.

### 3.8. Integrated Analysis of the lncRNA-Associated ceRNA Network

**Figure 8:**
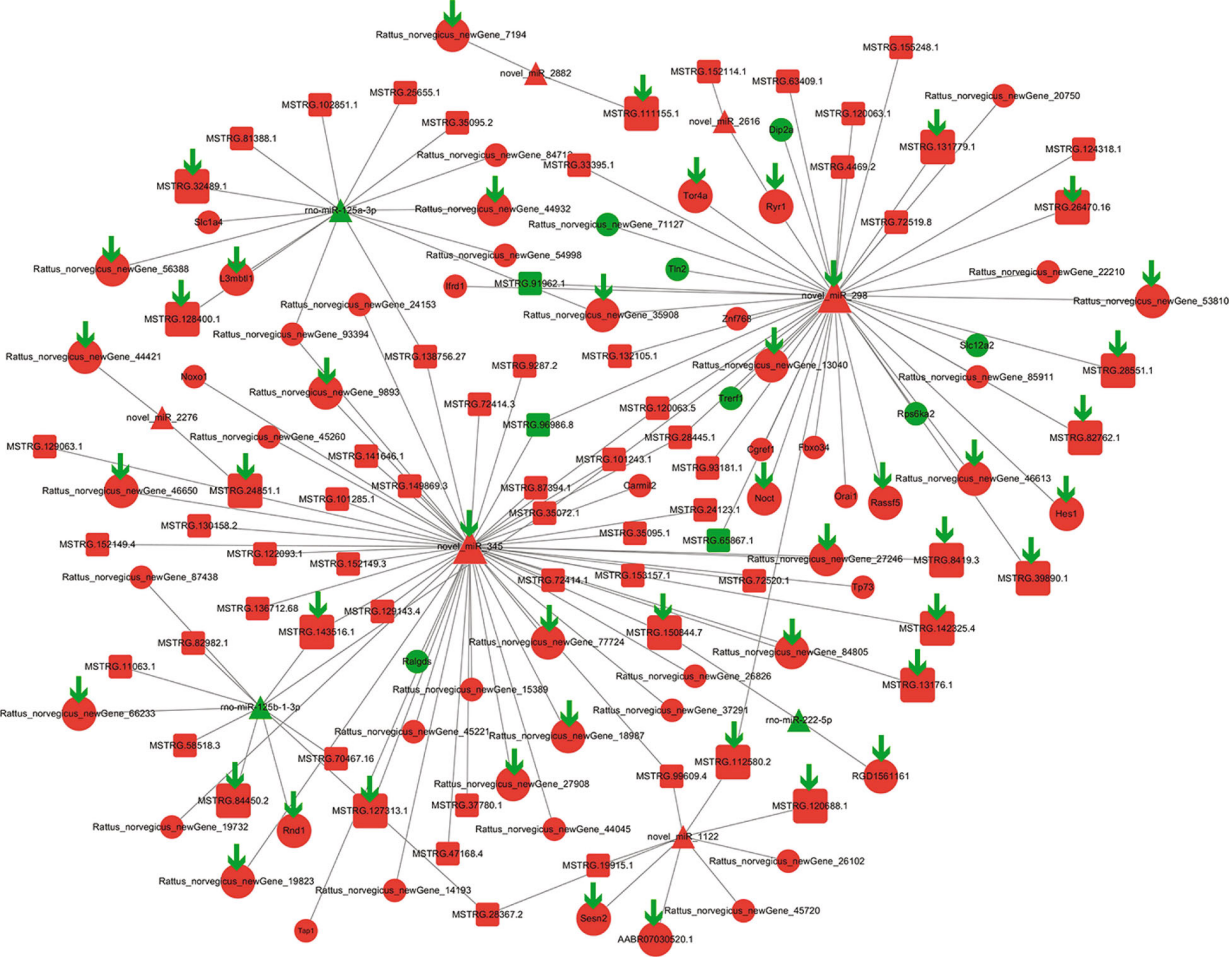
The interaction network of lncRNA–miRNA–mRNA. Circle, square, and triangle represent mRNA, lncRNA, and miRNA, respectively. Red and green represent upregulation and downregulation, respectively. The arrow represents the RNAs which QUE altered, and the downward direction indicates downregulation. lncRNA: long noncoding RNA; mRNA: messenger RNA; miRNA: microRNA; QUE: quercetin.

In this study, LncBase v.2 available in DIANA Tools was used to retrieve information on miRNA binding to specific lncRNAs, and Ingenuity Pathway Analysis was applied to ascertain the experimentally validated miRNA-to-mRNA interactions. Based on the DE RNAs identified between the control group and the model group, the lncRNA-associated ceRNA network related to OS (containing 65 mRNAs, 77 lncRNAs, and nine miRNAs) was constructed (Figure 8). Furthermore, treatment with QUE can act on the expression of 18 lncRNAs and subsequently regulate that of 27 mRNAs, by competitively binding with novel_miR_345 and novel_miR_298. The arrows in this lncRNA–miRNA–mRNA network represent RNAs that QUE acted on. Overall, the evidence obtained from our bioinformatics analysis suggests that lncRNAs harbour miRNA response elements and play pivotal regulatory roles in the antioxidant stress mechanism of QUE.

## 4. Discussion

To the best of our knowledge, this is the first comprehensive study of a lncRNA-associated ceRNA network to reveal regulator pathways with regard to the protective effect of QUE on neural cells from OS-induced damages. Our findings indicated that pretreatment with QUE markedly increased cell viability in a dose-dependent manner versus that observed for cells treated with H_2_O_2_ alone. In addition, we found that pretreatment with QUE induced the production of antioxidant enzymes and inhibited apoptosis in H_2_O_2_-induced PC-12 cells. Moreover, 197 DE lncRNAs, 14 DE miRNAs, and 294 DE mRNAs were identified as related to the antioxidant activities of QUE, and the results of sequencing were validated by real-time qPCR. The evidence obtained from the KEGG enrichment function analysis suggests that the protective effect of QUE may be related to the PI3K/AKT signalling pathway, which was further verified by western blotting. Based on authoritative databases, the lncRNA-associated ceRNA network was visualized for the comprehensive analysis of miRNAs, lncRNAs, and mRNAs. This approach could shed new light on the prevention of neurodegenerative disorders.

Firstly, we focused on the DE coding genes. A total of 966 H_2_O_2_-responsive genes (i.e., 823 upregulated and 143 downregulated) were detected. In addition, 495 QUE-responsive genes (i.e., 130 upregulated and 365 downregulated) were identified. By comparing the gene expression of the three groups, we finally determined that 294 mRNAs were potentially related to the protective effect of QUE against OS in PC-12 cells. Of those, 175 genes were newly discovered genes, and further follow-up studies are warranted for their functional annotation. Regarding other genes that have been previously annotated, some are involved in the mechanism of OS: hes family bHLH transcription factor 1 (HES1), dual specificity phosphatase 1 (DUSP1), BTG antiproliferation factor 2 (BTG2), regulator of G protein signalling 2 (RGS2), and activating transcription factor 3 (ATF3). HES1-mediated promotion of the extracellular matrix protein expression inhibits the proliferative and migratory functions of trabecular meshwork cells under OS [28]. Overexpression of DUSP1 increases cellular susceptibility to oxidative injury [29]. It has been suggested that the upregulation of BTG2 expression in response to OS may involve the ROS-protein kinase C-nuclear factor *κΒ* cascade [30]. Similarly, RGS2 has been implicated in OS, and a time- and concentration-dependent upregulation in RGS2 mRNA has been observed in human astrocytoma 1321N1 cells treated with H_2_O_2_ [31, 32]. Upregulation of ATF3 has been observed in H_2_O_2_-stimulated NP31 cells, as well as endothelial cells of the glomerulus and aorta of diabetic model rats [33]. Similar results were found in the present study. Elevated levels of HES1, DUSP1, BTG2, RGS2, and ATF3 were identified in the H_2_O_2_-treated group compared with the control group. Interestingly, the expression of these genes declined to control levels after pretreatment with QUE. The specific roles of these mRNAs associated with the antioxidative effect of QUE in neurodegenerative disease warrant further investigation.

The negative or positive role of lncRNAs is observed in the oxidation/antioxidant system [34, 35]. Therefore, studies aiming to explore the lncRNAs in the OS field may be helpful for the further investigation of specific biomarkers of OS-related diseases [18]. Antioxidant drugs that target lncRNA potentially provide a novel strategy for the treatment of neurodegenerative diseases. In our findings, a total of 1,186 lncRNAs were identified to be related to H_2_O_2_-induced OS, and a large number of lncRNAs were previously functionally characterized. Furthermore, a total of 197 lncRNAs were found to play critical roles in the protective effects of QUE on H_2_O_2_-induced oxidative injury. However, there is limited knowledge regarding the potential functions of these dysregulated lncRNAs. Future studies will contribute to the precise mechanism of lncRNAs in the protective effects of QUE against oxidative injury.

ROS are upstream regulators or downstream effectors of miRNAs, and both are inextricably linked to neurodegenerative processes [19]. Previous studies demonstrated that free radicals may be one of the signals that change the levels of miRNAs and their target proteins, leading to neurodegeneration in neurodegenerative diseases. Moreover, antioxidants that reduce OS may be affecting the levels of miRNAs [36, 37]. In the present study, a total of 135 miRNAs were observed in response to OS, and a limited number of miRNAs were found to play important roles in the antioxidant effects of QUE. Interestingly, all these miRNAs associated with the antioxidant properties of QUE were newly discovered. Further observations are needed to reveal the annotations of these miRNAs in neurodegenerative disease and their specific role in the antioxidant mechanism of QUE.

KEGG pathway analysis was performed to further predict the potential functions of the DE mRNAs and ncRNAs identified in this study. The pathway analysis on mRNAs indicated the crucial role of the FOXO signalling pathway related to the antioxidant mechanism of QUE. Furthermore, PLK2, MDM2, IL6, GADD45G, and CDKN1A in the FOXO signalling pathway were validated using real-time qPCR. In addition, pathway analysis of the miRNA target genes and lncRNA target genes related to QUE revealed that both are involved in the PI3K/AKT signalling pathway. Western blotting demonstrated that the levels of p-PI3K, p-AKT, PI3K, and AKT were increased in a dose-dependent manner by treatment with QUE. Manipulation of PI3K/AKT/FOXO3a signalling modulates OS, which may potentially provide novel therapeutic avenues for PD and AD [38]. It has been documented that actions on the PI3K/AKT pathway may be an important strategy associated with protecting cortical neurons against 4-hydroxynonenal-induced protein oxidation, lipid peroxidation, and mitochondrial dysfunction [39]. FOXO3a, an important downstream target of PI3K/AKT, was recognized as a protective factor that participates in controlling cell fate. OS has been found to trigger FOXO3a nuclear translocation and directly induce apoptosis. Moreover, klotho protein and resveratrol protect neurons from OS damage by increasing the phosphorylation of the PI3K/AKT/FOXO3a pathway [40–42]. Considering previous studies and our experimental results, we hypothesized that QUE could protect PC-12 cells from H_2_O_2_-induced oxidative damage by regulating the PI3K/AKT/FOXO3a pathway; however, this hypothesis requires further investigation.

RNA transcripts and lncRNAs with miRNA response elements may act as ceRNA to suppress miRNA function using shared miRNA response elements for mutual regulation [20]. Thus far, there are several ceRNA-related research studies investigating OS [22, 23]. In this study, we generated a putative lncRNA–miRNA–mRNA network describing the mechanism through which lncRNAs act as ceRNAs. This mechanism could potentially regulate mRNA expression by modulating the levels of its upstream miRNA regulators. Certain lncRNAs, acting as sponges involved in the protective effect of QUE against OS, were identified. Based on the results, 18 QUE-targeted lncRNAs could competitively bind to novel_miR_345 and novel_miR_298 and subsequently regulate the expression of 27 mRNAs. Of note, additional observations are warranted to further elucidate this coexpression network.

In conclusion, changes in the coding and noncoding transcriptomes of PC-12 cells and H_2_O_2_-induced PC-12 cells with and without QUE intervention were identified. By combining these results with those obtained from the bioinformatics analysis, this study indicates that aberrantly expressed miRNA, lncRNA, and mRNA participate in several specific biological processes and are involved in pathways related to OS and the antioxidant effect of QUE. Several limitations should be overcome in future studies aiming to more accurately reflect the antioxidant stress mechanism of QUE. ncRNA and mRNA changes in other cell and animal models of OS injury should also be examined. Additionally, further validation research is necessary to observe potential changes in the apoptotic rate of H_2_O_2_-induced PC-12 cells by manipulating the expression of top ncRNA and mRNA candidates.

## Figures and Tables

**Table 1:** Primers designed for qRT-PCR validation of candidate miRNAs, mRNAs, and lncRNAs.

Names	Forward primer and reverse primer	Tm (°C)	Product length (bp)
ACTB	F: 5′ CGAGTACAACCTTCTTGCAGC3′ R: 5′ ACCCATACCCACCATCACAC3′	60	202
*β*-Actin (R)	F: 5′CGAGTACAACCTTCTTGCAGC3′ R: 5′ ACCCATACCCACCATCACAC3′	60	202
U6	F: 5′GCTTCGGCAGCACATATACTAAAAT3′ R: 5′CGCTTCACGAATTTGCGTGTCAT3′	60	89
IL6	F: 5′ TCAGAGCAATACTGAAACCCTA3′ R: 5′ TCCTTAGCCACTCCTTCTGT3′	60	134
PLK2	F: 5′ ATGGTGGCGATCTCCCTAGT3′ R: 5′ AGCGAACAGCCAGACATCAA3′	60	247
MDM2	F: 5′ TGAGGTCTATCGGGTCACAGTC3′ R: 5′ CAGGCACATCTAAGCCTTCTTCT3′	60	263
CDKN1A	F: 5′ TCTTGTGATATGTACCAGCCACAG 3′ R: 5′ GTCAAAGTTCCACCGTTCTCG 3′	60	182
GADD45G	F: 5′ GTCTACGAGTCCGCCAAAGTC3′ R: 5′ CTATGTCGCCCTCATCTTCT3′	60	92
MSTRG.89940.1	F: 5′ GTCACCTTACCGTAGGCACA 3′ R: 5′ CCAAATTCCTCCAGCTTCACT 3′	60	157
MSTRG.4076.1	F: 5′ AGTGCTCCCGACATTCACTT 3′ R: 5′ TCCTTACGCTCAATCTATCCC 3′	60	103
MSTRG.48686.2	F: 5′ GCAGTACAACAATTATCGCCAA 3′ R: 5′ AGGTCCTTAGAGTGAGCAACG 3′	60	197
MSTRG.34031.1	F: 5′ TTTCCACAGCCTTTCCACG 3′ R: 5′ CACTTCCACATTGCTCTTCATC 3′	60	196
Novel_miR_1502	GSP: 5′GGGGAATACTGGGTGCTGT3 ′ R: 5′GTGCGTGTCGTGGAGTCG3′	60	64
Novel_miR_2218	GSP: 5′GGGGTTCTGGGTGCTGTA3′ R: 5′GTGCGTGTCGTGGAGTCG3′	60	62
Novel_miR_298	GSP: 5′GAAACGGCGGCGATG3′ R: 5′GTGCGTGTCGTGGAGTCG3′	60	59
Novel_miR_345	GSP: 5′GGAGGAGGGAACGCAGTCT3′ R: 5′GTGCGTGTCGTGGAGTCG3′	60	64
Novel_miR_97	GSP:5′GGGGGTGTCTGTCTGAGTG3′ R: 5′GTGCGTGTCGTGGAGTCG3′	60	62

## Data Availability

The data that support the findings of this study are available from the corresponding author upon reasonable request.
